# Supplementary material for: Gentrification and Air Quality in a Large Urban County in the United States
Source: Int J Environ Res Public Health. 2023 Mar 8;20(6):4762. doi: 10.3390/ijerph20064762 (PMC10049340; doi:10.3390/ijerph20064762)
Supplement: Supplementary file 1 [file ijerph-20-04762-s001.zip › ijerph-2234913-supplementary.pdf]

International Journal of
Environmental Research and Public Health

MDPI

Table S1. Median levels of pollutants in Wayne County based on gentrification and location from 1980 to 2020.

Air Pollutants[1]	Year				
	1980	1990	2000	2010	2020
Intense Gentrification (Median)					
VOCs	--	2.458	2.196	0.972	1.534
SO_2	7.121	7.755	4.984	2.700	1.061
$PM_{2.5}$	--	--	15.154	9.643	8.570
PM_{10}	29.630	33.344	28.638	19.716	17.716
Ozone	0.022	0.027	0.029	0.034	0.032
NO_2	27.654	20.240	19.125	10.086	10.703
Lead	0.303	0.065	0.037	0.039	0.010
CO	1.338	0.767	0.402	0.335	0.308
HAP	0.085	0.066	0.883	0.802	0.439
NOx	21.053	17.472	18.055	12.647	8.938
Gentrification (Median)					
VOCs	--	2.450	2.096	0.975	1.561
SO_2	8.768	7.789	5.260	2.720	0.997
$PM_{2.5}$	--	--	16.107	9.966	8.786
PM_{10}	29.525	34.302	29.284	19.633	17.582
Ozone	0.022	0.027	0.029	0.034	0.031
NO_2	26.785	20.658	19.722	10.230	10.881
Lead	0.287	0.064	0.038	0.026	0.009
CO	1.353	0.758	0.407	0.337	0.304
HAP	0.090	0.062	0.512	0.816	0.394
NOx	22.294	17.854	18.689	12.633	9.236
Non-Gentrified (Median)					
VOCs	--	2.458	2.343	0.943	1.520
SO_2	7.863	7.701	5.173	2.722	0.989
$PM_{2.5}$	--	--	15.802	9.974	8.679
PM_{10}	29.611	33.649	28.588	19.601	17.580
Ozone	0.022	0.027	0.029	0.034	0.031
NO_2	27.068	20.290	19.508	10.237	10.688
Lead	0.290	0.064	0.039	0.026	0.010
CO	1.341	0.764	0.408	0.337	0.307
HAP	0.088	0.064	0.499	0.812	0.403
NOx	21.514	17.680	18.692	12.684	9.144

[1] VOCs are measured in parts per million. SO_2 is measured in parts per million. $PM_{2.5}$ and PM_{10} are measured in micrograms per cubic meter. Ozone is measured in parts per billion. NO_2 is measured in parts per billion. Lead is measured in nanograms per cubic meter. CO is measured in parts per million. HAP are measured in parts per million. NOx are measured in parts per billion.
